# Artificial intelligence-assisted precise preoperative prediction of lateral cervical lymph nodes metastasis in papillary thyroid carcinoma via a clinical-CT radiomic combined model

**DOI:** 10.1097/JS9.0000000000002267

**Published:** 2025-02-04

**Authors:** Junze Du, Xingyun He, Rui Fan, Yi Zhang, Hao Liu, Haoxi Liu, Shangqing Liu, Shichao Li

**Affiliations:** a Department of Breast and Thyroid Surgery, Southwest Hospital, Army Medical University, Chongqing, China; b Department of Radiology, Southwest Hospital, Army Medical University, Chongqing, China; c Yizhun Medical AI, Beijing, China; d Department of Breast and Thyroid Surgery, Guiqian International General Hospital, Guiyang, China; e College of Medical Informatics, Chongqing Medical University, Chongqing, China

**Keywords:** CT radiomics, lateral cervical lymph nodes metastasis, papillary thyroid carcinoma

## Abstract

**Objectives:**

This study aimed to develop an artificial intelligence-assisted model for the preoperative prediction of lateral cervical lymph node metastasis (LCLNM) in papillary thyroid carcinoma (PTC) using computed tomography (CT) radiomics, providing a new noninvasive and accurate diagnostic tool for PTC patients with LCLNM.

**Methods:**

This retrospective study included 389 confirmed PTC patients, randomly divided into a training set (*n* = 272) and an internal validation set (*n* = 117), with an additional 40 patients from another hospital as an external validation set. Patient demographics were evaluated to establish a clinical model. Radiomic features were extracted from preoperative contrast-enhanced CT images (venous phase) for each patient. Feature selection was performed using analysis of variance and the least absolute shrinkage and selection operator algorithm. We employed support vector machine, random forest (RF), logistic regression, and XGBoost algorithms to build CT radiomic models for predicting LCLNM. A radiomics score (Rad-score) was calculated using a radiomic signature-based formula. A combined clinical-radiomic model was then developed. The performance of the combined model was evaluated using the receiver operating characteristic (ROC) curve, calibration curve, and decision curve analysis (DCA).

**Results:**

A total of 1724 radiomic features were extracted from each patient’s CT images, with 13 features selected based on nonzero coefficients related to LCLNM. Four clinically relevant factors (age, tumor location, thyroid capsule invasion, and central cervical lymph node metastasis) were significantly associated with LCLNM. Among the algorithms tested, the RF algorithm outperformed the others with five-fold cross-validation on the training set. After integrating the best algorithm with clinical factors, the areas under the ROC curves for the training, internal validation, and external validation sets were 0.910 (95% confidence interval [CI]: 0.729–0.851), 0.876 (95% CI: 0.747–0.911), and 0.821 (95% CI: 0.555–0.802), respectively, with DCA demonstrating the clinical utility of the combined radiomic model.

**Conclusions:**

This study successfully established a clinical-CT radiomic combined model for predicting LCLNM, which may significantly enhance surgical decision-making for lateral cervical lymph node dissection in patients with PTC.

## Introduction

**Highlights**

- Innovative clinical-radiomic model for precise preoperative LCLNM prediction in PTC.
- This study includes detailed analysis of 429 PTC cases with CT imaging data.
- 13 significant radiomic features selected via LASSO and machine learning techniques.
- Our model provides non-invasive decision support for precise thyroid cancer surgery.

Thyroid cancer is the seventh most common cancer worldwide, with a significant increase in incidence over the last four decades^[1]^. Papillary thyroid carcinoma (PTC) accounts for approximately 80% of cases and presents a high incidence of early lymph node metastasis, affecting 30%–80% of patients, with around 40% demonstrating lateral cervical lymph node metastasis (LCLNM)^[2]^. The primary treatment is surgical intervention, often followed by iodine-131 therapy and thyroid stimulating hormone (TSH) suppression. However, excessive surgical procedures can lead to postoperative neck pain and sensory loss, affecting quality of life and increasing complication rates, while inadequate surgery raises the risk of recurrence and reoperation, with complications potentially escalating by 5–10 times^[3–6]^. Despite a generally favorable prognosis, around 13.3% of PTC patients experience recurrence, predominantly in cervical lymph nodes^[7,8]^. Patients with LCLNM have higher local recurrence rates and poorer overall survival compared to those with only central cervical lymph node metastasis (CCLNM), even after therapeutic dissection and high-dose radioactive iodine ablation, which can increase morbidity and reduce quality of life^[9,10]^. Therefore, thorough preoperative evaluation of LCLNM is essential for optimizing surgical strategies and improving patient outcomes.

The American Thyroid Association (ATA) guidelines emphasize the importance of preoperative evaluation of both the primary tumor and cervical lymph nodes to guide surgical decisions^[11]^. Preoperative neck ultrasound (US) and US-guided fine-needle aspiration (FNA) biopsy are primary diagnostic tools for detecting lymph node metastasis in PTC patients^[12]^. However, the diagnostic performance of neck US varies among practitioners, with interobserver variability highest in assessing LCLNM^[13,14]^, A meta-analysis of 16 studies involving 3044 patients reported a low pooled sensitivity of US for predicting LCLNM at 70%^[15]^. Computed tomography (CT) imaging can significantly reduce missed diagnoses of LCLNM, improving surgical planning^[16]^. Despite its advantages, the full potential of CT scans in the preoperative evaluation of PTC patients is often underutilized, as CT provides crucial insights into the primary tumor and its relationship with surrounding tissues, including lymph node metastasis, which is vital for determining the extent of lymph node dissection.

Radiomics, utilizing artificial intelligence (AI) techniques, extracts quantitative features from medical images to reflect tumor heterogeneity and predict outcomes noninvasively, offering a novel approach to precision medicine^[17,18]^. While there have been reports on using radiomics techniques based on US^[19]^ or CT imaging^[20]^ to predict CCLNM, the application of radiomics for assessing LCLNM remains limited. By combining clinical risk factors with CT radiomics, we aim to refine the decision-making process for lateral lymph node dissection in PTC patients, in line with the principles of precision surgery.

This study proposes to develop a clinical-CT radiomic model to predict LCLNM in PTC patients, providing evidence-based support for precise surgical treatment planning.

## Methods

### Patients and study design

Ethical approval for this study was granted by the Research Ethics Committee (No: KY2024108) on 29 March 2024, in accordance with the Declaration of Helsinki.

We retrospectively collected data from patients with PTC treated at the first center from January 2021 to December 2022, while external validation included patients from another center during the same period.

Inclusion criteria were: (1) patients with PTC who underwent initial surgical treatment with pathological confirmation; (2) patients who underwent lateral cervical lymph node dissection (LCLND) due to FNA biopsy-confirmed LCLNM or suspicious preoperative US results, with complete clinical data; (3) CT scans performed within 1 month prior to surgery; (4) no prior treatment for thyroid-related diseases.

Exclusion criteria were: (1) other organ malignancies; (2) multifocal PTC; (3) history of thyroid or cervical lymph node surgery, or acute/chronic inflammation; (4) unclear imaging or incomplete clinical data; (5) severe cardiac, liver, or renal dysfunction.

**Figure 1.**
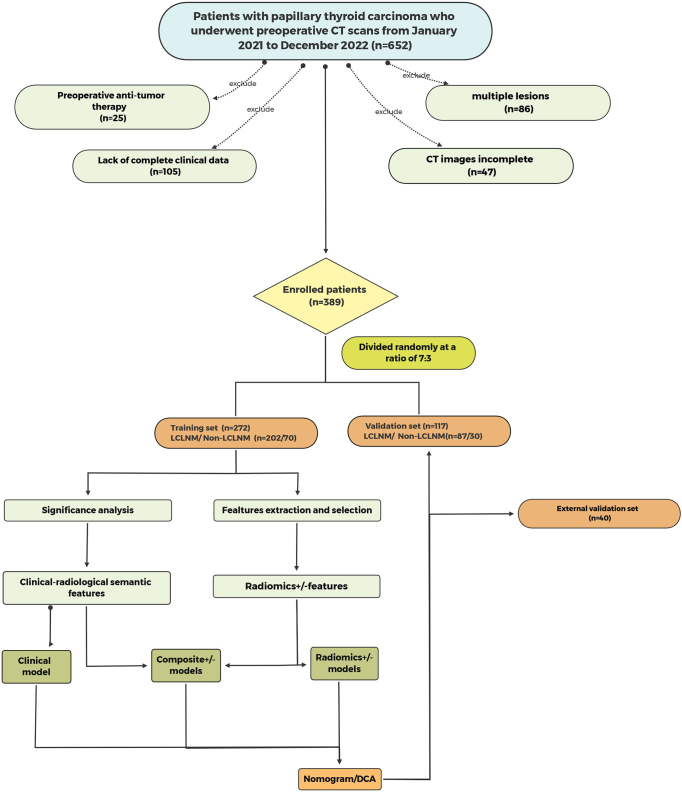
Flowchart for patient selection.

Enrolled patients were randomly divided into a training cohort (*n* = 272) and a testing cohort (*n* = 117) in a 7:3 ratio, with an additional 40 patients for external validation. The study flow diagram is shown in Figure 1. This study was conducted and reported according to the STROCSS 2021 Guideline (Detailed information can be found in Supplemental files)^[21]^. Collected clinical data included age, gender, disease duration, body mass index, tumor size and location, CCLNM, thyroid capsule invasion, and various thyroid function tests and antibodies.

### Construction of the clinical model

Univariate and backward stepwise multivariate logistic regression (LR) analyses (using minimum Akaike information criterion (AIC) criteria) were employed to evaluate the significance of clinical factors in predicting LCLNM in the training set. A *P*-value of less than 0.05 was deemed significant for model construction. Odds ratios with 95% confidence intervals (CIs) were calculated for each independent factor.

### CT image acquisition and segmentation

CT scans were obtained using either a 64-slice spiral CT scanner (Siemens Somatom Definition AS, Germany) or a dual-source spiral CT scanner (Somatom Definition Flash, Germany). Scanning parameters included a tube voltage of 100–120 kV, automatic tube current adjustment, and a pitch of 1.2–1.5. For contrast-enhanced scans, a nonionic contrast agent (iohexol, iodine 300 mg/mL) was administered at a dose of 80–100 mL and an injection rate of 3.5–4.0 mL/s. A circular region of interest (ROI) of approximately 1 cm^2^ was selected within the ascending aorta, with continuous axial scanning starting 10 s post-contrast injection. The arterial phase scan was conducted 5 s after reaching a CT value of 120 HU, followed by a venous phase scan 15–20 s later.

**Figure 2.**
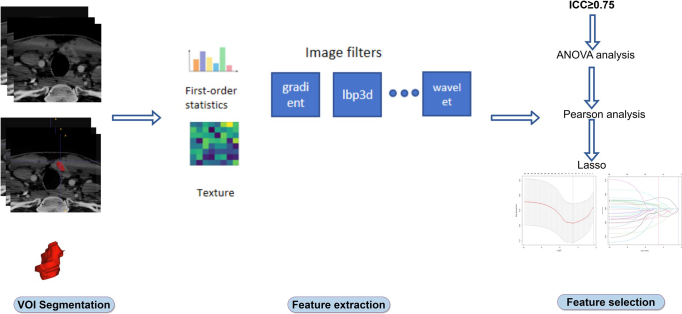
CT image segmentation. VOI, volume of interest.

Data were uploaded to a big data AI platform (Shenrui Medical Technology, Beijing), where a semiautomatic method delineated the ROIs on venous phase images. Two radiologists with over 10 years of experience outlined the ROIs, being aware of the thyroid cancer diagnosis but blinded to clinical and histopathological data. One month later, ROIs for 30 randomly selected patients were redrawn by both radiologists, and intraclass correlation coefficients (ICCs) were calculated to assess the consistency of tumor characterization (Fig. 2).

## Radiomics analysis

### Feature extraction

Pyradiomics (version 3.0.1), an open-source Python package^[22]^, was utilized to extract radiomic features in accordance with the Image Biomarker Standardization Initiative guidelines^[23]^. Each ROI of PTC CT lesions underwent feature extraction, with details provided in Table S1 of the appendix, http://links.lww.com/JS9/D801. Images were resampled to a target spacing (determined by the median spacing of all patient Digital Imaging and Communications in Medicine (DICOM) images) using B-spline interpolation, which preserves tissue contrast differences effectively^[24]^. Seven filters (exponential, gradient, square, square root, logarithm, lbp2D, and lbp3D) were applied to both original and derived images for each patient. In addition, eight sets of wavelet-transformed images were generated using various combinations of high-pass and low-pass filters. In total, 1724 radiomic features were extracted from 16 imaging sets (1 original, 8 wavelet, and 7 filtered). The main categories of radiomic features included: (1) first-order statistics based on pixel gray-level histograms; (2) shape metrics; (3) texture metrics derived from gray-level co-occurrence matrix, gray-level dependence matrix (GLDM), gray-level run-length matrix, and neighboring gray-tone difference matrix. To assess segmentation robustness, 50 patients were randomly selected for multi-radiologist segmentation, with ROIs segmented twice by two radiologists. For feature preprocessing, non-informative features with zero variance were discarded, and Z-score normalization was applied to each feature.

### Feature selection

Feature stability was assessed using ICC, retaining features with high stability (ICC ≥ 0.75) for further analysis. A three-step approach was employed to select the optimal radiomic features for classification. First, analysis of variance (ANOVA) was used to identify significant features, retaining those with a *P*-value < 0.05. Second, redundant features with Pearson correlation coefficients exceeding 0.90 were removed. Finally, the Lasso method was applied to select the most informative features, using 10-fold cross-validation to determine the optimal parameter λ over 5000 iterations. The Lasso method, based on this optimal λ, calculated coefficients for each feature, with nonzero coefficients indicating the selected features.

### Development of machine learning-based radiomics models

The first data were divided into a training set and an internal validation set in a 7:3 ratio, and model selection and parameter tuning were performed on the training set using five-fold cross-validation. Four commonly used machine learning models (SVM, RF, LR, and XGBoost) were employed to construct the models. Due to the severe class imbalance in both set (with a class ratio of 1:3), Synthetic Minority Oversampling Technique^[25]^ was carried out on the training set to balance the class distribution during the training process. In addition, the area under the curves (AUC), balanced accuracy, sensitivity, specificity, calibration curves, and decision curve analysis (DCA) were used to evaluate each model. The DeLong test was used to compare the values of AUC in different models. The Python scikit-learn package (version 0.21) was used to establish and evaluate the model^[26]^.

## Model interpretation

The SHAP package^[27]^ regards all the features as “contributors” and generates a SHAP value. The SHAP value diagram and variable importance graph are used to show the contribution and importance ranking of each feature to the model, respectively.

## Construction of the clinical radiomics nomogram

A clinical radiomics nomogram was developed by integrating significant clinical features with the Rad-score. Calibration curves were used to assess the nomogram’s calibration. The diagnostic performance of the clinical model, the radiomics signature model, and the clinical radiomics nomogram was evaluated based on AUC values from both the training and testing cohorts. To assess the clinical effectiveness of the nomogram, DCA was conducted, calculating the net benefit across the threshold probability range in both cohorts.

## Statistical analysis

Basal statistical tests, along with univariate and backward stepwise multivariate LR analyses, were conducted using the “autoReg”^[28]^ and “StepReg”^[29]^ packages, feature heatmaps were plotted using the “ComplexHeatmap” package^[30]^, and correlation heatmaps were drawn using the “linkET” package^[31]^ in R (v4.2.2) on the RStudio platform (v2023.03.0-386). Radiomics feature standardization, selection, and statistical analyses were performed on the Anaconda3 platform using Python 3.6, primarily employing the “scikit-learn”^[26]^ and “matplotlib”^[32]^ packages. Continuous variables were presented as the mean ± standard deviation. The independent sample t-test or Mann–Whitney *U* test was used to compare continuous variables. The chi-square test or Fisher’s exact test was used for categorical variables. A two-sided *P* value of less than 0.05 was considered statistically significant.

## Results

### Clinical features of the patients and construction of the clinical model

**Figure 3.**
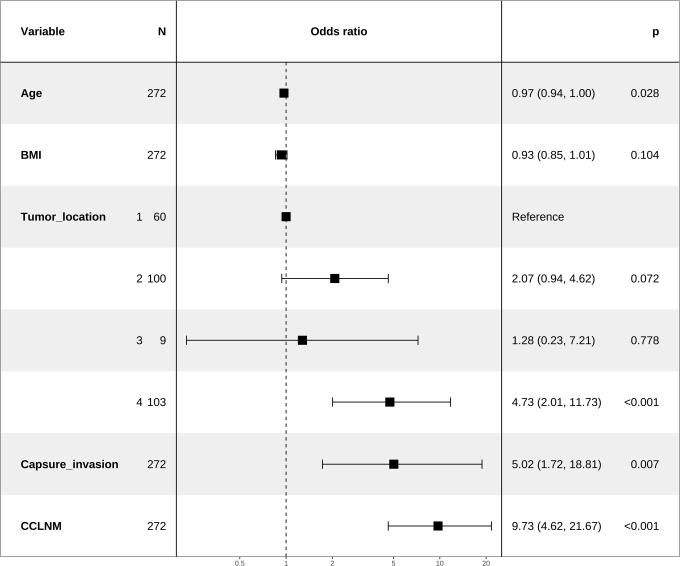
Selected clinical features correlated with LCLNM after univariate and backward stepwise multivariate logistic regression analyses: 1, lower; 2, middle; 3, isthmus; 4, upper.

A total of 389 patients with confirmed PTC were enrolled from the first center. According to a 7:3 ratio, 272 of them were randomly divided into the training set (202/70, LCLNM/non-LCLNM), and the rest 117 were divided into the internal validation set (87/30, LCLNM/non-LCLNM). As an independent external cohort, 40 patients from another hospital were enrolled in the validation set (23/17, LCLNM/non-LCLNM). The clinical characteristics of both LCLNM and non-LCLNM groups in the training and validation sets are summarized in Table 1. Logistic regression analysis identified four factors significantly associated with LCLNM: age, tumor location in the upper thyroid, thyroid capsule invasion, and CCLNM (Fig. 3). A clinical prediction model was constructed based on these four factors. In addition, 31 patients exhibited skip metastasis to LCLNM without CCLNM involvement.

**Table 1.** Clinical features of the enrolled patients in training set and validation set

Clinical factors	Traning set			Internal validation set			External validation set		
	LCLNM	Non_LCLNM	*P*-value	LCLNM	Non_LCLNM	*P*-value	LCLNM	Non_LCLNM	*P*-value
	(*N* = 202)	(*N* = 70)		(*N* = 87)	(*N* = 30)		(*N* = 23)	(*N* = 17)	
Age	40.41 ± 11.65	45 ± 11.87	0.006	43 ± 12.03	42 ± 11.43	0.685	44.65 ± 14.28	38 ± 12.85	0.131
Gender			0.823			0.912			0.216
Male	53 (26.24%)	20 (28.57%)		29 (33.33%)	9 (30.00%)		5 (21.74%)	1 (5.88%)	
Female	149 (73.76%)	50 (71.43%)		58 (66.67%)	21 (70.00%)		18 (78.26%)	16 (94.12%)	
Course	7.61 ± 16.17	7.89 ± 10.87	0.871	10.24 ± 27.8	4.41 ± 8.48	0.086	15.6 ± 34.11	6.86 ± 11.34	0.261
BMI	23.71 ± 3.6	24.82 ± 3.87	0.038	24.55 ± 4.38	25.14 ± 4.07	0.501	24.6 ± 4.09	22.44 ± 2.21	0.039
Tumor size	1.78 ± 1.07	1.65 ± 2.79	0.705	1.89 ± 1.14	1.48 ± 1.62	0.206	1.44 ± 1.32	1.36 ± 0.77	0.824
Tumor location			0.016			<0.001			0.014
Isthmus	4 (1.98%)	5 (7.14%)		1 (1.15%)	1 (3.33%)		2 (8.70%)	0 (0.00%)	
Upper	83 (41.09%)	20 (28.57%)		36 (41.38%)	3 (10.00%)		11 (47.83%)	2 (11.76%)	
Middle	77 (38.12%)	23 (32.86%)		38 (43.68%)	12 (40.00%)		5 (21.74%)	4 (23.53%)	
Lower	38 (18.81%)	22 (31.43%)		12 (13.79%)	14 (46.67%)		5 (21.74%)	11 (64.71%)	
Capsule invasion			0.019			0.626			0.12
Negative	165 (81.68%)	66 (94.29%)		70 (80.46%)	26 (86.67%)		8 (34.78%)	11 (64.71%)	
Positive	37 (18.32%)	4 (5.71%)		17 (19.54%)	4 (13.33%)		15 (65.22%)	6 (35.29%)	
Hashimoto thyroiditis			0.501			0.372			0.712
Negative	135 (66.83%)	43 (61.43%)		54 (62.07%)	22 (73.33%)		16 (69.57%)	10 (58.82%)	
Positive	67 (33.17%)	27 (38.57%)		33 (37.93%)	8 (26.67%)		7 (30.43%)	7 (41.18%)	
Thyroid nodules			0.491			1			0.675
Negative	160 (79.21%)	52 (74.29%)		68 (78.16%)	24 (80.00%)		15 (65.22%)	13 (76.47%)	
Positive	42 (20.79%)	18 (25.71%)		19 (21.84%)	6 (20.00%)		8 (34.78%)	4 (23.53%)	
T3	2.16 ± 1.54	2.01 ± 0.43	0.217	2.06 ± 0.61	2.05 ± 0.48	0.942	1.76 ± 0.24	2.06 ± 0.48	0.028
T4	109.35 ± 20.24	108.35 ± 20.85	0.729	108.67 ± 27.95	106.5 ± 29.82	0.729	92.83 ± 33.98	95.14 ± 26.95	0.812
FT3	5.53 ± 3.46	5.06 ± 1.01	0.081	5.11 ± 2.15	5.06 ± 1.02	0.876	5.02 ± 0.57	5.07 ± 0.83	0.862
FT4	17.14 ± 2.39	16.97 ± 2.52	0.615	17.07 ± 4.79	17.24 ± 3.33	0.832	17.33 ± 3.1	16.05 ± 2.41	0.15
TSH	2.32 ± 1.72	2.26 ± 1.29	0.767	2.4 ± 2.04	2.55 ± 1.79	0.718	6.24 ± 14.25	2.6 ± 1.22	0.235
TG	52.68 ± 105.87	34.2 ± 68.53	0.097	37.41 ± 92.35	26.4 ± 39.14	0.369	105.16 ± 168.08	23.77 ± 29.74	0.032
TGAb	211.82 ± 544.87	767.88 ± 5649.18	0.414	423.21 ± 2731.76	59.37 ± 86.73	0.218	181.91 ± 728.8	162.13 ± 288.13	0.907
TMAb	10.49 ± 10.37	9.79 ± 10.27	0.626	10.25 ± 9.78	8.57 ± 7.47	0.331	10.49 ± 12.93	9.36 ± 8.02	0.736
TPOAb	49.16 ± 91.9	51.27 ± 76.95	0.851	42.69 ± 73.41	49.83 ± 59.36	0.596	50.93 ± 106.73	97.98 ± 191.86	0.371
CCLNM			<0.001			<0.001			0.03
Negative	20 (9.90%)	32 (45.71%)		11 (12.64%)	19 (63.33%)		3 (13.04%)	8 (47.06%)	
Positive	182 (90.10%)	38 (54.29%)		76 (87.36%)	11 (36.67%)		20 (86.96%)	9 (52.94%)	

TSH, thyroid stimulating hormone.

### Feature extraction and selection

A total of 1724 quantitative radiomic features were extracted from CT images. After excluding features with poor reproducibility (ICCs < 0.75) and stability, 446 features were selected for further analysis using ANOVA and Pearson correlation. The least absolute shrinkage and selection operator method ultimately identified 13 key radiomic features for building the radiomic model (Fig.4A, B). The 13 retained features and their corresponding weight coefficients are displayed in Figure 4C. These features belong to six categories based on image information and computational methods: Shape Features, Wavelet Transform Features, Local Binary Pattern, Run Length Matrix Features, Gray Level Dependence Matrix Features, and Neighbouring Gray Tone Difference Matrix Features. We explored correlations between the 13 radiomic features and four clinical factors (age, capsule invasion, CCLNM, LCLNM), but these correlations were weak. Tumor location showed no significant correlation with any of the 13 features (Fig. 4D). These 13 features are distributed between the two groups (LCLNM and non-LCLNM) as shown in Figure 4E.

**Figure 4.**
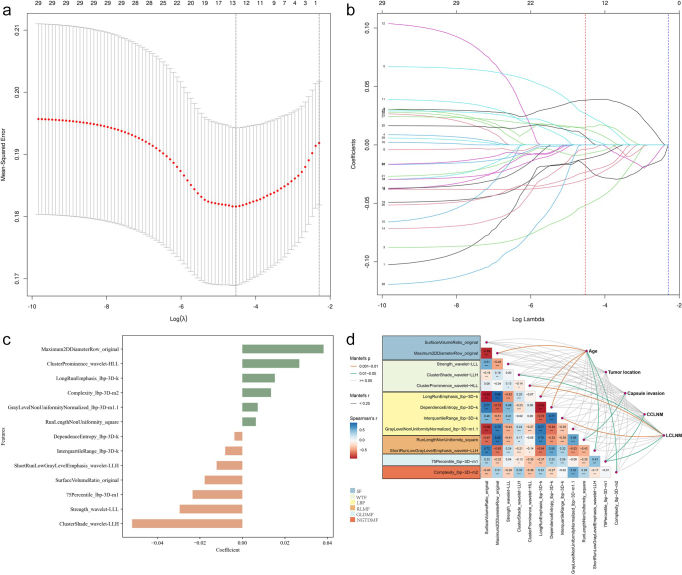

**Figure 4.**
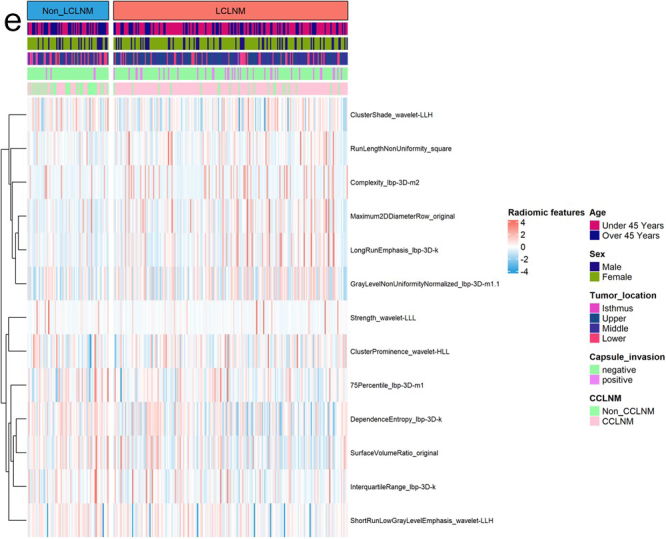
The process of radiomic feature extraction and selection. (A) Lasso coefficient path of radiomic features. (B) Cross-validation curve for feature selection process. (C) The 13 final retained radiomic features and their corresponding weight coefficients. (D) Heatmap of 13 final radiomic features for prediction of LCLNM. Each row corresponds to an individual patient in training cohort and each column corresponds to each scaled radiomic feature. The color key outlines the corresponding radiomic features. Clinical parameters including age, sex, tumor location, capsule invasion, and CCLNM are represented. (E) The correlation heatmap of the 13 radiomic features and their correlations with clinical factors. SF, shape features; WTF, wavelet transform features; LBP, local binary pattern; RLM, run length matrix; GLDM, gray level dependence matrix; NGTDM, neighboring gray-tone difference matrix.

### Machine-learning radiomic model

Among four classical machine-learning algorithms, the RF model demonstrated the best performance in the training set with five-fold cross-validation (as shown in Table S2, Appendix, http://links.lww.com/JS9/D801). Therefore, RF was selected as the final radiomic model. To minimize overfitting, we fine-tuned the RF model’s hyperparameters using a grid search, with a restricted search space for each parameter. The model’s performance on the training, internal validation, and external validation sets is presented in Table 2.

**Table 2.** Results of the models based on enhanced CT imaging

Model	Metrics	Balanced_accuracy	AUC	Specificity	Sensitivity	F1 score
Radiomics model	Training set	0.689 (0.644–0.724)	0.857 (0.804–0.900)	0.406 (0.340–0.468)	0.971 (0.928–1.000)	0.573 (0.504–0.634)
	Internal validation set	0.634 (0.556–0.712)	0.781 (0.680–0.875)	0.402 (0.297–0.505)	0.867 (0.741–0.970)	0.556 (0.444–0.652)
	External validation set	0.665 (0.515–0.813)	0.711 (0.524–0.872)	0.565 (0.360–0.773)	0.765 (0.556–0.944)	0.65 (0.457–0.810)
Clinical model	Training set	0.656 (0.597–0.715)	0.786 (0.597–0.715))	0.941 (0.907–0.971)	0.371 (0.258–0.486)	0.872 (0.838–0.903)
	Internal validation set	0.737 (0.646–0.824)	0.836 (0.646–0.824)	0.908 (0.844–0.958)	0.567 (0.390–0.739)	0.883 (0.830–0.926)
	External validation set	0.662 (0.523–0.794)	0.733 (0.523–0.794)	0.913 (0.773–1.000)	0.412 (0.188–0.650)	0.778 (0.640–0.885)
Clinincal-radiomics model	Training set	0.794 (0.729–0.851)	0.910 (0.729–0.851)	0.931 (0.896–0.963)	0.657 (0.529–0.763)	0.908 (0.876–0.936)
	Internal validation set	0.832 (0.747–0.911)	0.876 (0.747–0.911)	0.931 (0.872–0.978)	0.733 (0.565–0.885)	0.920 (0.878–0.959)
	External validation set	0.684 (0.555–0.802)	0.821 (0.555–0.802)	0.957 (0.850–1.000)	0.412 (0.182–0.636)	0.800 (0.654–0.897)

## Model interpretability

**Figure 5.**
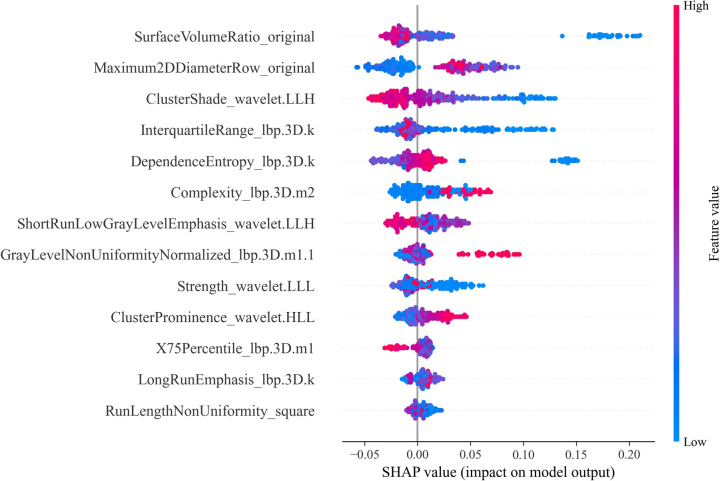
SHAP plot of the 13 important radiomic features.

The SHAP plot (Fig. 5) illustrates the contribution of each radiomic feature in predicting LCLNM within the training set. The color gradient, ranging from blue to red, reflects the increasing absolute value on the horizontal axis from left to right. A negative value on the horizontal axis, with a larger absolute magnitude, suggests a higher probability of a negative predictive outcome. Conversely, a positive value with a larger magnitude indicates a higher probability of a positive outcome. For example, a larger Maximum2DDiameterRow_original increase the likelihood of lymph node metastasis, while a higher ClusterShade_wavelet.LLH decreases this likelihood. Among all features, SurfaceVolumeRatio_original is identified as the most significant predictor.

### Establishment and validation of a clinical radiomics model

**Figure 6.**
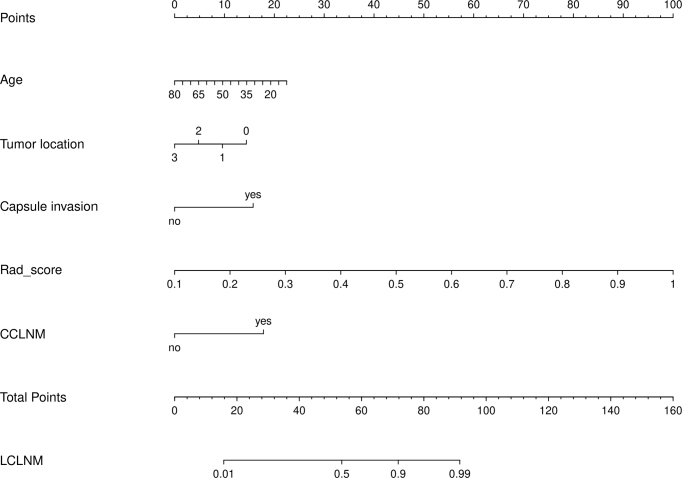
The clinical-CT radiomic nomogram. The values of the Rad-score and clinical factors can be converted into quantitative values according to the points axis. After summing the individual points to achieve the final sum shown on the total points axis, the prediction of LCLNM in PTC before surgery was performed.

The AUC for the clinical model were 0.786 (95% CI: 0.597–0.715), 0.836 (95% CI: 0.646–0.824), and 0.733 (95% CI: 0.523–0.794) in the training, internal and external validation set, respectively. In comparison, the AUC for the radiomic model were 0.857 (95% CI: 0.804–0.900), 0.781 (95% CI: 0.680–0.875), and 0.711 (95% CI: 0.524–0.872) in the training, internal and external validation set, respectively. To further improve prediction accuracy, a nomogram combining the clinical model with radiomic model was developed (Fig. 6). The final combined model demonstrated competitive AUC values of 0.910 (95% CI: 0.729–0.851), 0.876 (95% CI: 0.747–0.911), and 0.821 (95% CI: 0.555–0.802) in the training, internal and external validation set, respectively, demonstrating a strong discriminatory ability (Fig. 7A–C).

**Figure 7.**
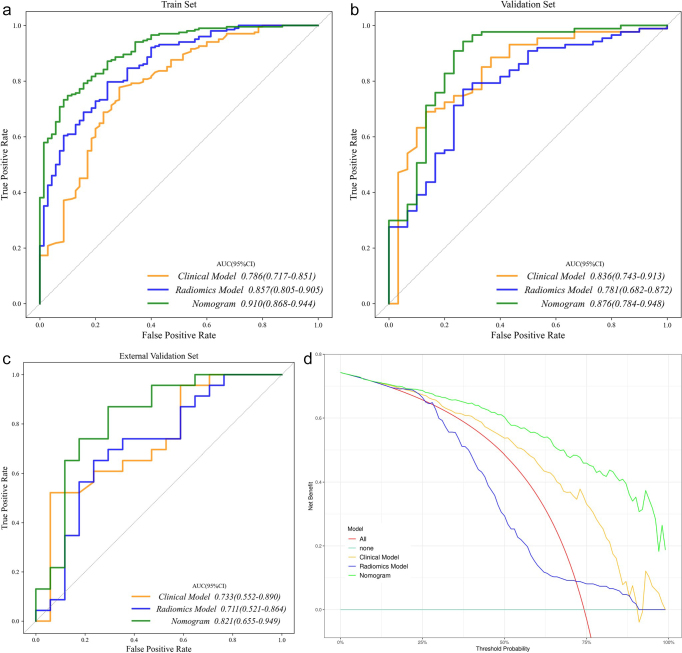

**Figure 7.**
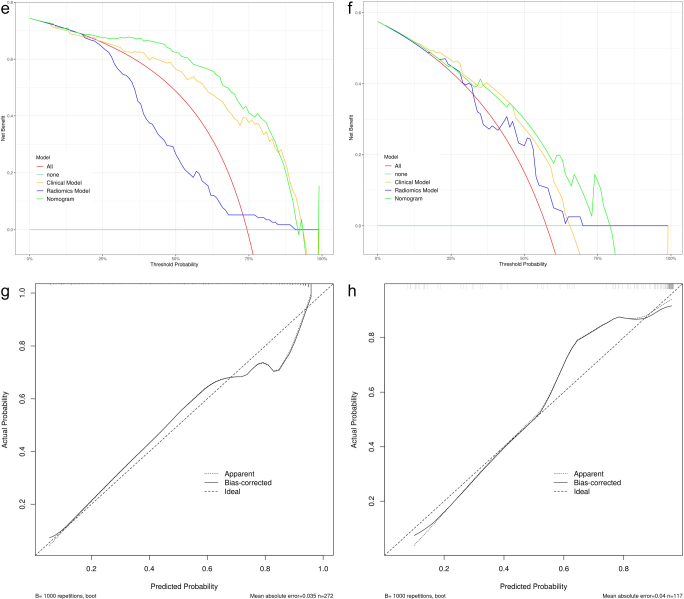

**Figure 7.**
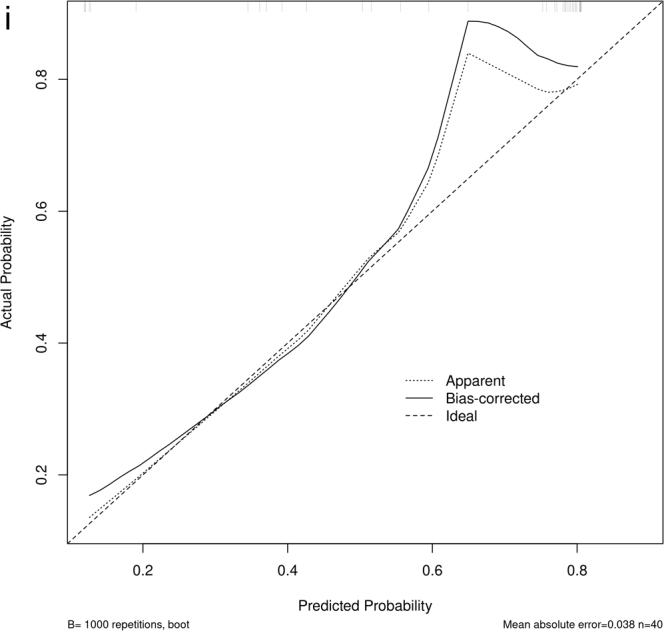
ROC and DCA curves for the clinical model, radiomic model, and clinical-radiomics model. The calibration curves of the clinical-radiomics models in the training set, internal validation set and external set respectively. (A) ROC curves of the three models in the training set. (B) ROC curves of the three models in the internal validation set. (C) ROC curves of the three models in the external validation set. (D) DCA curves of the three models in the training set. (E) DCA curves of the three models in the internal validation set. (F) DCA curves of the three models in the external validation set. (G) Calibration curves for the clinical-radiomics model in the training set. (H) Calibration curves for the clinical-radiomics model in the internal validation set. (I) Calibration curves for the clinical-radiomics model in the external validation set.

**Figure 8.**
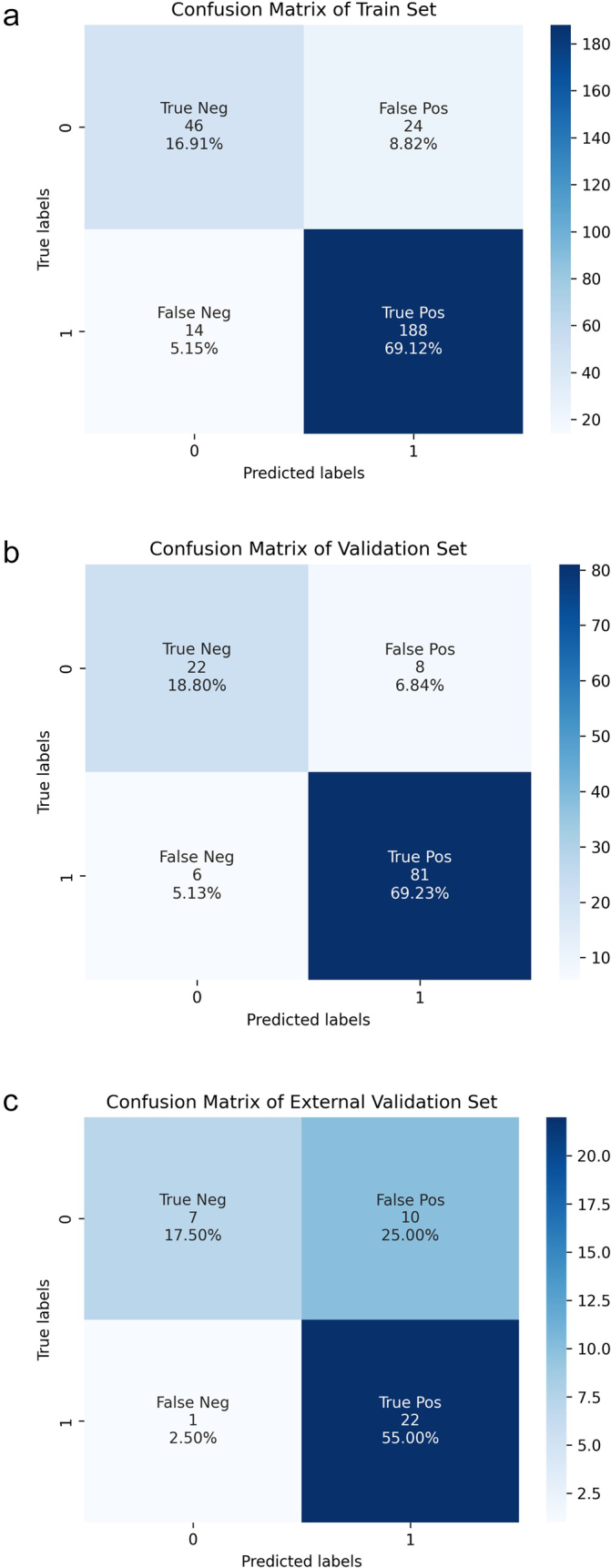
The confusion matrix of the clinical-radiomics model. (A) Training set. (B) Internal validation set. (C) External validation set.

DCA showed that this combined model provided a significant net clinical benefit across all sets (Fig. 7D–F). Calibration curves for the clinical-radiomics model plotted using the R package “rms”^[33]^ with 1000 bootstrap samples, showed good agreement between predicted and observed outcomes, with the curves closely aligned with the ideal calibration line (Fig. 7G–I). In addition, the confusion matrix highlighted low false-positive rates in validation sets (Fig. 8), indicating that the model effectively identifies patients with LCLNM and minimizes the risk of overtreatment. A detailed comparison of the three models is provided in Table 2. Given the class imbalance, balanced accuracy was reported to ensure more accurate assessment across the sets.

### Translation to clinical application

To facilitate clinical application, this model is now available for free online at AI Models for PTC(https://sqliu.shinyapps.io/AI_Models_PTC_R/).

## Discussion

Cervical lymph node metastasis is a common pattern in PTC, making cervical lymph node dissection crucial. The choice between central cervical lymph node dissection alone or in combination with LCLND significantly influences the patient’s treatment, follow-up, and prognosis^[34]^. In PTC, local lymph node metastasis typically occurs in a specific order: from perithyroidal and paratracheal nodes (level VI) to the upper mediastinum, jugular chain, and beyond (levels VII, IV, III, II, V, I), eventually leading to distant sites such as the lungs.

There is significant debate regarding the management of cervical lymph node metastasis, particularly concerning prophylactic cervical lymph node dissection. Although advancements in preoperative imaging techniques, such as US, CT, MRI, and FNA cytology, allow for the detection of CCLNM and LCLNM in most patients, some patients remain classified as cN_0_, where preoperative assessment is inconclusive. Research indicates that 50%–60% of cN_0_ patients harbor metastatic foci^[35]^. Prophylactic LCLND is generally not recommended for this group. Current consensus suggests that intraoperative rapid pathology can be utilized in cN_0_ PTC cases to determine the necessity of LCLND based on central compartment findings. However, some studies advocate for LCLND in cN0 patients for more accurate postoperative staging and reduced recurrence and metastasis rates^[36,37]^. In addition, a notable proportion of PTC patients exhibit skip metastasis, where LCLNM occurs without central lymph nodes involvement, with reported incidences ranging from 0.6% to 37.5%^[38]^. In this study, among 389 patients, 31 (7.9%) cases displayed skip metastasis, consistent with previous findings.

However, some researchers oppose the prophylactic dissection of lateral lymph nodes due to its complexity, potential complications, and impact on patients’ quality of life^[39,40]^. The central issue in this debate is the accurate preoperative identification of LCLNM. Therefore, reliably determining LCLNM in PTC before surgery is crucial for developing effective surgical strategies, optimizing tumor staging, predicting local recurrence, and tailoring individual treatment plans, ultimately enhancing patient outcomes.

Among the 389 PTC patients in this study, postoperative pathology confirmed LCNM in 289 (74.29%) cases, while 100 (25.71%) cases were free of LCNM. The relatively high incidence of patients without LCNM suggests potential overtreatment, which may stem from reliance on US as the primary imaging modality for surgical planning. Although the ATA guidelines endorse US as the first-line noninvasive assessment tool for cervical lymph node status, studies indicate it has high specificity (80.5%–97.4%) but lower sensitivity (36.7%–61.0%) in preoperative evaluations^[41]^. To enhance the accuracy of surgical decision-making, this study investigated the effectiveness of four machine learning models based on CT radiomics to predict LCNM in PTC and aimed to identify the optimal diagnostic model. In addition, the study identified age, tumor location, tumor capsule invasion, and CCLNM as independent risk factors for LCNM. The nomogram model built using the best algorithm combined with clinical risk factors achieved AUC values of 0.910 (95% CI: 0.729–0.851) for the training set and 0.876 (95% CI: 0.747–0.911) for the internal validation set. While previous radiomic studies on PTC have primarily focused on US analyses, such as Tong *et al’s* evaluation of US radiomics for predicting LCLNM which reported an AUC of 0.946 with 21 features^[42]^. US has inherent limitations. In contrast, CT scans provide comprehensive three-dimensional views of fixed, large, and retrosternal lesions, and enhanced CT more sensitively and clearly visualizes small lymph nodes, enabling metastasis assessment based on enhancement patterns^[16]^. Although some studies have employed enhanced CT for radiomic analyses of primary PTC lesions to predict CCLNM^[43–45]^, they typically have small sample sizes and focus on CCLNM. This study, however, addresses the prediction of LCLNM with a larger sample size, facilitating a more comprehensive analysis of the radiomic characteristics in the lateral cervical region and supporting precise regional lymph node dissection in PTC.

In developing this predictive model, we classified the 13 radiomic features into six groups based on image information and computational methods. We analyzed their correlation with four clinical factors, revealing weak correlations between age, tumor capsule invasion, CCLNM, and LCLNM with certain features; however, tumor location showed no correlation with any of the features. Radiomic features reflect various biological and physical properties within the images, potentially linked to tumor heterogeneity, growth patterns, cellular density, blood flow, and the tumor microenvironment. A comprehensive multi-omics approach integrating genomics, transcriptomics, proteomics, metabolomics, pathomics, and spatial omics could enhance understanding of the relationships between radiomic features and tumor biology.

This study aimed to achieve accurate staging while minimizing overtreatment. The ATA guidelines recommend FNA when suspicious LCLNM is identified on imaging, with therapeutic LCLND required when LCLNM is confirmed. However, FNA is an invasive, time-consuming, and labor-intensive procedure that can be costly and is unsuitable for micronodules smaller than 1 cm^[46]^. Our model may enhance radiologists’ diagnostic capabilities, potentially reducing unnecessary FNAs in patients with PTC and aiding in determining the appropriate surgical scope. DCA and confusion matrix results indicate that the combined model can identify patients with LCLNM while effectively minimizing overtreatment.

The incidence of CCLNM is higher than that of LCLNM. Consequently, some studies have been published to predict the status of CCLNM based on US or CT imaging^[47–49]^. Our work primarily combined clinical risk factors with CT radiomics technology to construct a model for predicting the status of LCLNM. In the future, we plan to expand this model to jointly predict the metastasis status of both CCLNM and LCLNM, thereby further enhancing the preoperative decision-making efficacy of lymph node dissection in PTC.

There were still some limitations in the present study. First, although a retrospective study, including two centers and had an independent external validation set with limited cohort size, our model requires prospective further validation to confirm its diagnostic performance in real-world settings. Second, patients with multifocal PTC were excluded to prevent ambiguity regarding which lesion may have contributed to metastasis. In addition, genomic information was not incorporated into our model due to limited data from some patients. Existing literature indicates that individuals with the BRAFV600E mutation may have an increased risk of lymph node metastasis^[50,51]^. Therefore, we should integrate quantitative imaging features with genomic phenotypes and explore the biological interpretation based on the fusion model to make our model more understandable and valuable, and when we have a larger sample size dataset, we will validate our current findings in future studies.

## Conclusion

This study explored the use of AI technology to develop a clinical-CT radiomic model for predicting LCLNM in patients with PTC. This model has great potential to enhance surgical planning for LCLND. However, the findings are preliminary and based on retrospective cohorts, necessitating further validation through larger randomized controlled trials to confirm reliability and generalizability. While the results are promising, cautious interpretation is advised until additional evidence is available. Nonetheless, this research adds to the growing evidence that AI-assisted models may significantly impact precision medicine for PTC patients.

## Data Availability

The data that support the finding of this study are available from the Southwest Hospital, Army Medical University, but restrictions apply to the availability of these data, which were used under license for the current study and therefore not publicly available. However, data are available from the corresponding author, Shichao LI, upon reasonable request and with permission of the ethics committee of the Southwest Hospital, Army Medical University.

## Research registration unique identifying number (UIN)

The study received approval from the institutional review board of our institution (BIIT2024118). This study is a retrospective analysis and, as such, did not involve the recruitment of new participants or the collection of new data. According to standard research guidelines, retrospective studies that solely rely on the analysis of previously collected data do not fall under the requirement for clinical trial registration, Therefore, this study was not registered as a clinical trial.
